# Tuning the Structure of Pd@Ni–Co Nanowires and Their Electrochemical Properties

**DOI:** 10.1021/acs.jpclett.4c00376

**Published:** 2024-04-04

**Authors:** Dariusz Łukowiec, Magdalena Gwóźdź, Alina Brzęczek-Szafran, Tomasz Wasiak, Dawid Janas, Jerzy Kubacki, Stanisław Wacławek, Adrian Radoń

**Affiliations:** † Materials Research Laboratory, Faculty of Mechanical Engineering, Silesian University of Technology, Konarskiego 18A, Gliwice 44-100, Poland; ‡ Faculty of Chemistry, Silesian University of Technology, Krzywoustego 4, Gliwice 44-100, Poland; § August Chełkowski Institute of Physics, Faculty of Science and Technology, University of Silesia, 75 Pułku Piechoty 1, Chorzów 41-500, Poland; ∥ Institute for Nanomaterials, Advanced Technologies and Innovation, Technical University of Liberec, Studentská 1402/2, Liberec 1 461 17, Czech Republic; ⊥ Łukasiewicz Research Network, Institute of Non-Ferrous Metals, Sowińskiego 5, Gliwice 44-100, Poland

## Abstract

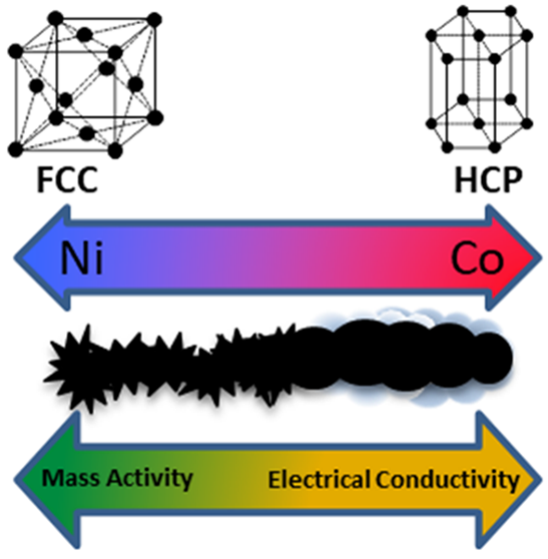

One-dimensional transition metal materials are promising supports for precious metals used in energy production processes. Due to their electrochemical properties, 3d-group metals (such as Ni, Co, and Fe) can actively interact with catalysts by a strong metal–support interaction. This study shows that changing the Ni:Co ratio makes it possible to modulate the structure of the catalyst supports, which, in turn, provides a tool for designing their electrical and electrochemical properties. For example, Ni_1_–Co_9_ shows the highest electrical conductivity (5.8–10^–4^ S/cm) among all of the materials examined. On the contrary, the Pd@Ni_7_–Co_3_ system presents the highest mass activity (>2000 mA mg^–1^) at 0.7 V, exceeding by several times that of commercial Pt/C (>300 mA mg^–1^) at the same potential. Our study opens the gateway for applications of bimetallic transition metal nanowires in catalytic conversion and energy production processes.

In the era of dependence on fossil fuels (responsible for producing 62% of the world’s electricity in 2020), there is a growing need for the utilization of energy sources that are safe for society and the surrounding environment. Fuel cells are one of the most promising solutions that can provide power for motor vehicles and electronic devices.^1−7^ The chemical energy stored in the molecules is converted into electrical energy by employing electrochemical systems with a dedicated catalyst. The resulting electricity does not impose any additional burden on the environment due to the near-zero emissions of carbon monoxide and NO_*x*_ compounds.^8−11^ Furthermore, additional advantages of fuel cells, as long as they have access to fuel and oxidant, are that they do not need to be recharged, as do Li-ion batteries, and they are safe to use.^12,13^ The main obstacle to improving the performance and lifetime of the cells is the poor kinetics of the electrochemical reactions, which is particularly evident in cathodic oxygen reduction reactions (ORRs). This is due to the high activation energy of the processes responsible for energy conversion, including O–O breakdown, the removal of oxidized products from the catalyst, multiple-electron transfer, and the various possible adsorption pathways of O_2_ molecules.^14,15^

Among the most efficient catalysts for the ORR, and thus the most commonly used, are systems based on Pt and Pd.^13^ However, the high price of these alloys combined with their low stability and lifetime severely limits their large-scale application. In contrast, another approach is to develop ORR catalysts without platinum-group metals (PGMs), and these include carbon materials, Fe-based systems,^16^ metal–organic framework materials,^17^ perovskite oxides,^18^ and pyrolyzed M-Nx.^19^ Despite their proven activity in ORRs, Pt-based systems are still far superior to them, but catalysts with high activity and stability can be achieved by alloying PGMs with transition metals such as Co, Fe, Ni, and Cu.^20^ Changing the catalyst’s electron structure (d-band center) and lattice parameters by alloying with another metal improves its catalytic properties.^21−23^ On the contrary, it brings a risk that, due to their different corrosion stability, one of the components of the bimetallic catalyst may degrade more rapidly.^24^ Further strategies involve optimizing the size (including single-atom catalysts)^25,26^ and shape of the metallic catalyst to expose catalytically active crystal facets.^27^

The use of transition metals or metal oxides as supports for catalyst nanoparticles can increase the yield of ORRs through a strong metal–support interaction (SMSI).^12,13^ The properties of transition metals, such as an oxygen adsorption capacity that is higher than that of Pt or a variable oxidation state, improve mass and electron transfer in ORRs.^13^ Via combination of a noble metal with a metal or metal oxide substrate, the local electron structure of the catalyst is modulated, often resulting in improved reaction kinetics. For instance, the combination of Pt with a metal oxide substrate (antimony–tin oxide) resulted in an improvement in the catalytic efficiency of the ORR.^28^ Moreover, combining a Pt catalyst with a CoO substrate also resulted in a >3-fold increase in bulk activity compared to that of commercial Pt/C.^29^ A Pd catalyst may substitute for Pt formulations due to the similar electronic and chemical properties while reducing the cost.^30−36^ The use of Pd in ORR processes is further supported by its greater tolerance to methanol poisoning in the event of crossover.^32,37^ In turn, pure Pd, compared to Pt, exhibits lower ORR catalytic activity and stability in acidic media.^38−43^ Recent results have indicated that this issue can be resolved by combining Pd with transition metals.^44^ Examples of joining a Pd catalyst with a metal/metal oxide substrate can also be found in the literature. Pd nanoparticles dispersed on a porous film of NiMnO gave a half-wave potential of 0.84 V in the ORR and a mass activity (MA) of 180.9 A g_Pd_^–1^.^45^ Even higher performance was observed when Pd/W_18_O_49_ was used as a support, showing an *E*_1/2_ of 0.932 V and a MA of 216 A g_Pd_^–1^, outperforming commercial Pd/C and Pt/C catalysts.^46^

In this study, we present the fabrication of bimetallic Ni–Co nanowires as supports for the Pd catalyst, taking advantage of the one-dimensional (1D) shape of the support that can provide enhanced electron transport and diffusion through the liquid environment in the ORR.^47,48^ By changing the concentrations of Ni and Co in the support material, we obtained systems with diverse structures. To the best of our knowledge, this is the first study that analyzes the effect of changing the structure of the bimetallic support on the electrochemical properties of the Pd catalyst in the ORR. To determine the role of the Pd–nanowire interaction, the supports before (Ni_*x*_–Co_*y*_) and after Pd deposition (Pd@Ni_*x*_–Co_*y*_) were studied. On the basis of this thorough characterization, we elucidated the structure–property relationships of Pd-doped Ni–Co nanowires in terms of providing guidelines for the future design of bimetallic supports for efficient ORRs.

The synthesis of the nanowires involved changing the concentration of Ni and Co metal precursors according to a scheme of 9:1, 8:2, 7:3, 6:4, 5:5, 3:7, and 1:9, followed by decorating the produced materials with Pd using a fixed precursor concentration of 0.5 mM. Transmission electron microscopy (TEM) in high-resolution (HRTEM) and scanning (STEM) modes and X-ray diffraction (XRD) techniques were used to characterize the structure and morphology of the materials obtained. Phase analysis of the synthesized Ni_*x*_–Co_*y*_ supports showed the presence of two main phases in their structure: face-centered cubic Ni(Co) and hexagonal Co_0.75_Ni_0.25_ (Figure 1c). At the same time, with an increase in the content of Ni in the material (Ni_6_–Co_4_, Ni_7_–Co_3_, and Ni_8_–Co_2_), the cubic structure of Ni(Co) prevailed, while a higher concentration of Co (Ni_1_–Co_9_) promoted crystallization of the hexagonal structure of Co_0.75_Ni_0.25_. The Ni(Co) phase appeared in the BF-DF STEM image as spiky particles from which the nanowires were composed (Figure 1a). The formation of the nanowires from the spherically shaped particles (Figure 1b) was related to the self-organization of the Co-rich hexagonal phase (Co_0.75_Ni_0.25_) under a magnetic field. Moreover, as shown in Figure 1b, the presence of the Co-rich hexagonal phase changed the nanowires’ morphology and influenced their surface oxidation process. The Pd@Ni_1_–Co_9_ nanowires synthesized using a high Co precursor concentration were covered by an oxidized plate-like structure (marked as a blue area in Figure 1b). In addition, no further changes were observed in the structure of bimetallic Ni–Co nanowires after the deposition of Pd nanoparticles on their surfaces (Figure 1d), showing that the metallic core of the nanowires was not affected by the decoration process. Furthermore, no reflections from Pd were observed in the X-ray patterns of the Pd@Ni_*x*_–Co_*y*_ materials, which was related to the detection limit of the technique used. Thus, inductively coupled plasma optical emission spectroscopy (ICP-OES) was used to confirm the presence and determine the concentration of Pd in the synthesized materials. It was observed that changing the phase composition in the samples affected the amount of deposited Pd on the surface of the support. Despite using the same K_2_PdCl_4_ precursor concentration (0.5 mM) for all of the analyzed supports, the amount of deposited Pd varied among the different materials (Table S1).

**Figure 1:**
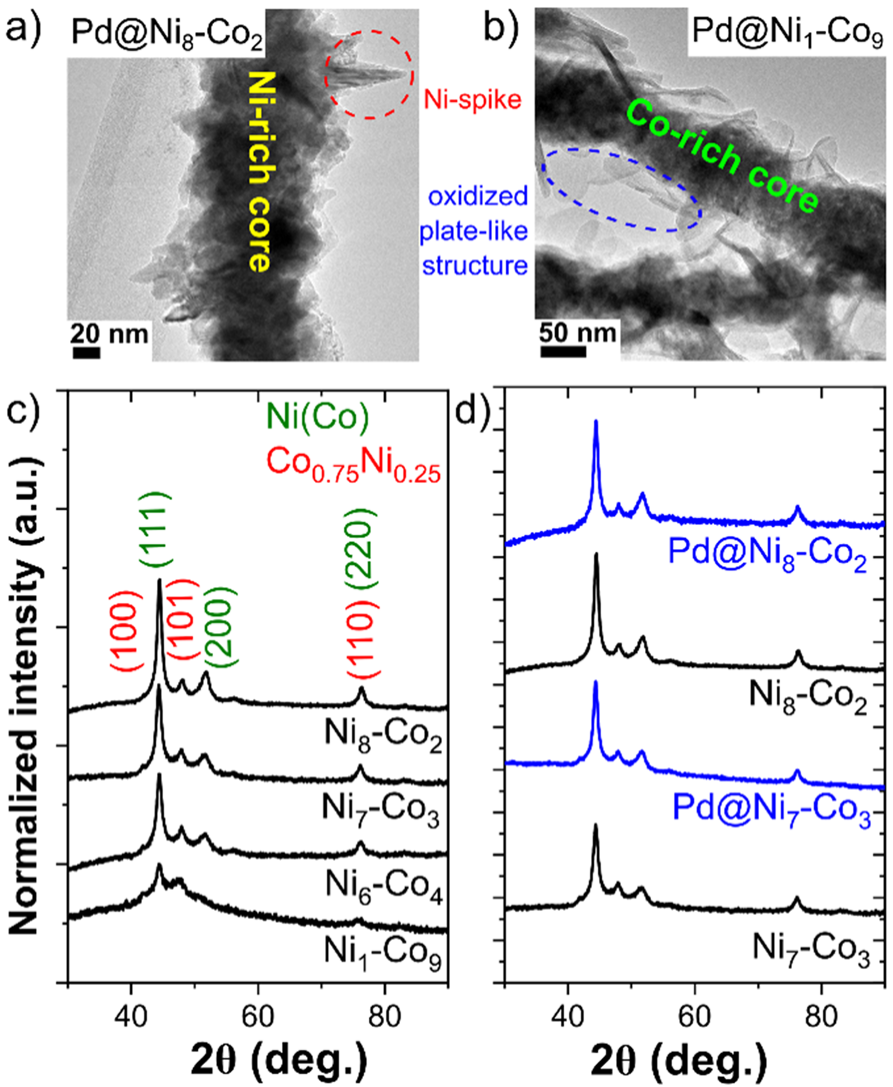
(a) BF-DF STEM image of Pd@Ni_8_–Co_2_. (b) BF-DF STEM image of Pd@Ni_1_–Co_9_. (c) XRD patterns of Ni_*x*_–Co_*y*_ nanowires with marked Miller indices of fcc Ni(Co) and hcp Co_0.75_Ni_0.25_ phases. (d) Comparison of XRD patterns of selected Ni_*x*_–Co_*y*_ nanowires before and after the Pd deposition process.

Through the use of TEM, the effect of phase composition on the morphology of the materials obtained was interpreted in detail. First, all of the analyzed materials were 1D nanowires with a diameter in the range of 40–80 nm on which Pd nanoparticles were deposited (Figure 2a–d and Figure S1a–f). However, the surface morphologies of the individual materials differed from each other, which was due to the changes in the ratio of the cubic to hexagonal phases. Materials with a predominant amount of Ni (Ni_7_–Co_3_, Ni_8_–Co_2_, and Ni_9_–Co_1_) and thus a cubic phase of Ni(Co) crystallized as needles on the surface of nanowires (Figure 2a,b and Figure S1a–d). In the case of materials with an increased concentration of Co (Ni_3_–Co_7_ and Ni_1_–Co_9_), the formed nanowires were spherical, which might be related to a higher concentration of the Co_0.75_Ni_0.25_ hexagonal phase (Figure 2c,d and Figure S1e,f). The differences mentioned above in the morphology of the obtained supports should offer different electric and electrochemical properties due to the dissimilar degrees of surface development and, furthermore, the number of available active centers of the catalyst.

**Figure 2:**
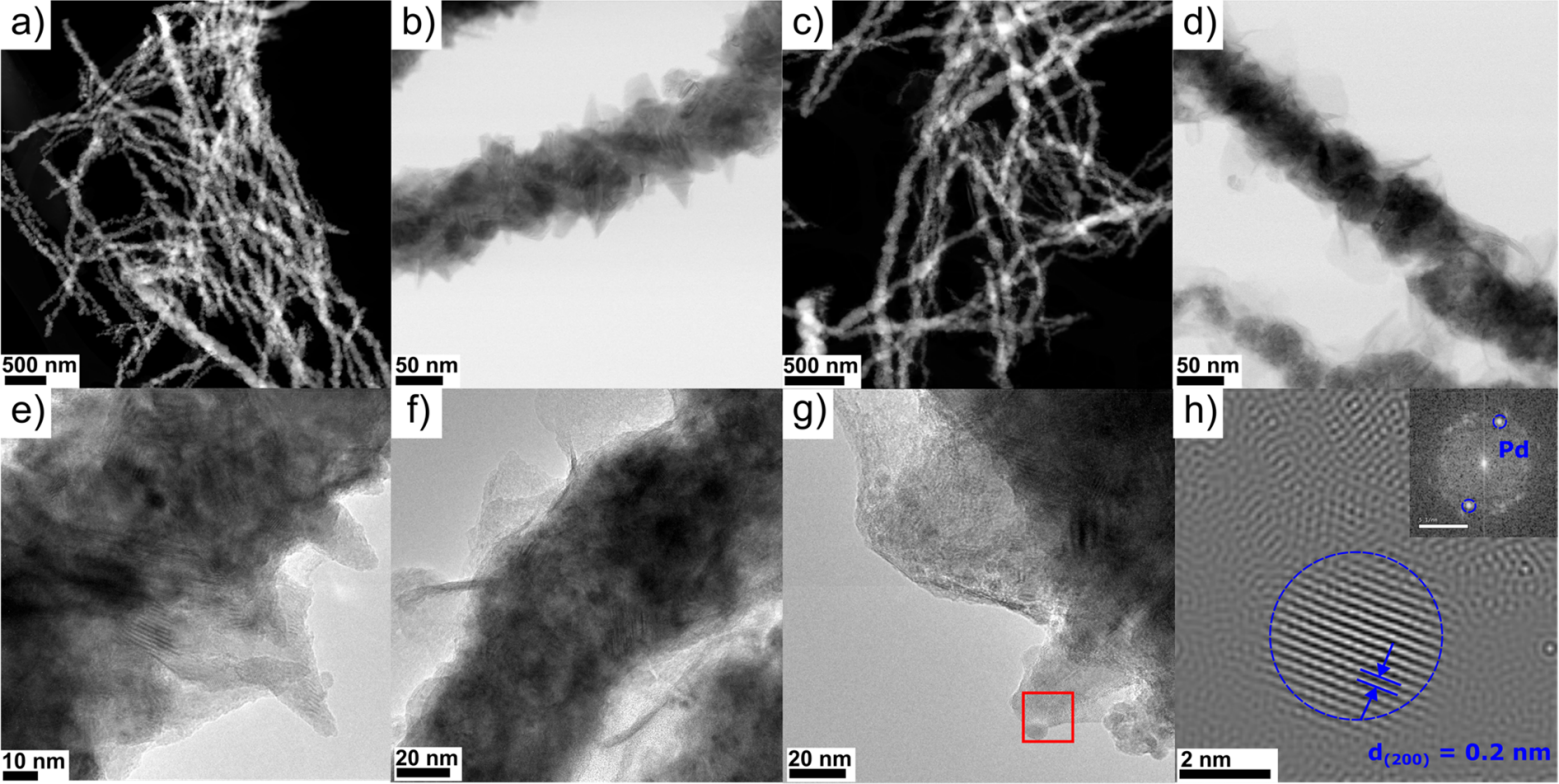
(a) HAADF-STEM and (b) BF-DF STEM images of Pd@Ni_8_–Co_2_. (c) HAADF-STEM and (d) BF-DF STEM images of Pd@Ni_3_–Co_7_. (e) HRTEM image of Pd@Ni_8_–Co_2_. (f) HRTEM image of Pd@Ni_1_–Co_9_. (g) HRTEM image of Pd@Ni_7_–Co_3_. (h) Enlarged image of the area boxed in red in panel g with identified interplanar distance characteristic for Pd (200) planes (the inset image shows the received fast Fourier transform with marked spots characteristic of the Pd phase).

TEM analysis revealed that the surfaces of the fabricated nanowires with a predominantly hexagonal phase (Pd@Ni_3_–Co_7_) were surrounded by a metallic oxide layer in the form of plates composed of Ni and Co oxides, which was confirmed by EDX (Figure S2). The core of the nanowires in the analyzed materials was metallic and consisted mainly of Ni and Co, which was further confirmed by XPS results as reported below. The small Pt peak on the spectrum from the Pd@Ni_3_–Co_7_ core came from H_2_PtCl_6_·6H_2_O, which was used as a nucleating agent in the synthesis of the nanowires.^49−52^ Thus, the type of dominant phase in the material [Ni(Co) or Co_0.75_Ni_0.25_] influenced not only the way the surface of the wires was formed into needles or spherical beads but also the occurrence of the surrounding oxide layer. HRTEM images of the Pd@Ni_8_–Co_2_ and Pd@Ni_7_–Co_3_ materials showed the surface of the support in the form of needles whose main phase was Ni(Co) (Figure 2e,g). In addition, the presence of spherical palladium nanoparticles with a diameter of ∼5 nm and a metallic nature [interplanar distance characteristic for Pd (200) planes], which were deposited on the surface of the Ni–Co support, was confirmed (Figure 2e,g,h). Analysis of the material with a majority of Co in the structure (Pd@Ni_1_–Co_9_) revealed that the hexagonal Co_0.75_Ni_0.25_ phase was responsible for the spherical nature of the support surface (Figure 2f). Meanwhile, the surface of the supports was surrounded by an oxide layer of Ni and Co, which was confirmed by XPS and EDX.

Studies of the electronic structure and electrical conductivity of the materials obtained were performed using spectroscopic techniques, X-ray photoelectron spectroscopy (XPS) and broadband dielectric spectroscopy (BDS), respectively. As part of the XPS studies, the chemical nature of the core was determined, as well as the degree of oxidation of the metallic layer’s elements. In addition, the chemical nature of the Pd nanoparticles deposited on the surface of the support was analyzed. The survey spectra of the Pd@Ni_7_–Co_3_, Pd@Ni_8_–Co_2_, and Pd@Ni_9_–Co_1_ samples showed mainly signals from Ni, Co, Pd, and O (Figure 3 and Figures S3–S5). The presence of an oxidized surface was detected in all of the analyzed samples, which was linked to the oxophilic nature of the Ni and Co constituting the support.^53−55^ The fabricated materials were also characterized after a short (1 min) etching with an Ar^+^-ion beam with an energy of 1 keV (Figures S3b–S5b). High-resolution spectra of the individual elements from Pd@Ni_8_–Co_2_, Pd@Ni_7_–Co_3_, and Pd@Ni_9_–Co_1_, before and after etching, are presented in Figure 3b–d and Figures S6, S7, S8a–f, S9a–f, and S10a–f. The Ni 2p_3/2_ photoemission line consisted of three components representing metallic Ni, NiO, and Ni_2_O_3_ located at binding energies of 852.9, 854.3, and 856.5 eV, respectively (Figure 3b).^47,56−58^ Ni_2_O_3_ exhibited the highest intensity on the recorded spectrum, although a short etching of the material indicated that the support core was primarily Ni^0^ (Figure S8d). Additional components located above 857 eV came from satellite structures of the main 2p_3/2_ line. The shape of the Co 2p_1/2_ line indicated the presence of four components, where the first, located at a binding energy of 793.8 eV, was metallic core Co, while the second came from Co_3_O_4_ at 797.5 eV.^59,60^ The other two were additional Co components and satellite structures (Figure 3c). Analysis of the Pd 3d_3/2_ and Pd 3d_5/2_ spectra provided information about the presence of Pd in the sample in the form of Pd^0^ and Pd^4+^. The recorded lines of metallic Pd and oxidized PdO_2_ were located at 335.9 and 337.6 eV, respectively (Figure 3d).^53,61^ Notably, for the Ni_8_–Co_2_@Pd sample, the intensity of the Pd^0^ line was higher, which reflected the facilitated crystallization toward metallic Pd. Similar characteristics of Pd^0^/Pd^4+^ were observed for Pd@Ni_7_–Co_3_ and Pd@Ni_9_–Co_1_, except that the further sample was distinguished by the highest PdO_2_ line intensity (Figures S9c and S10c). The atomic and weight concentrations of the cores of the tested samples are listed in Table S2.

**Figure 3:**
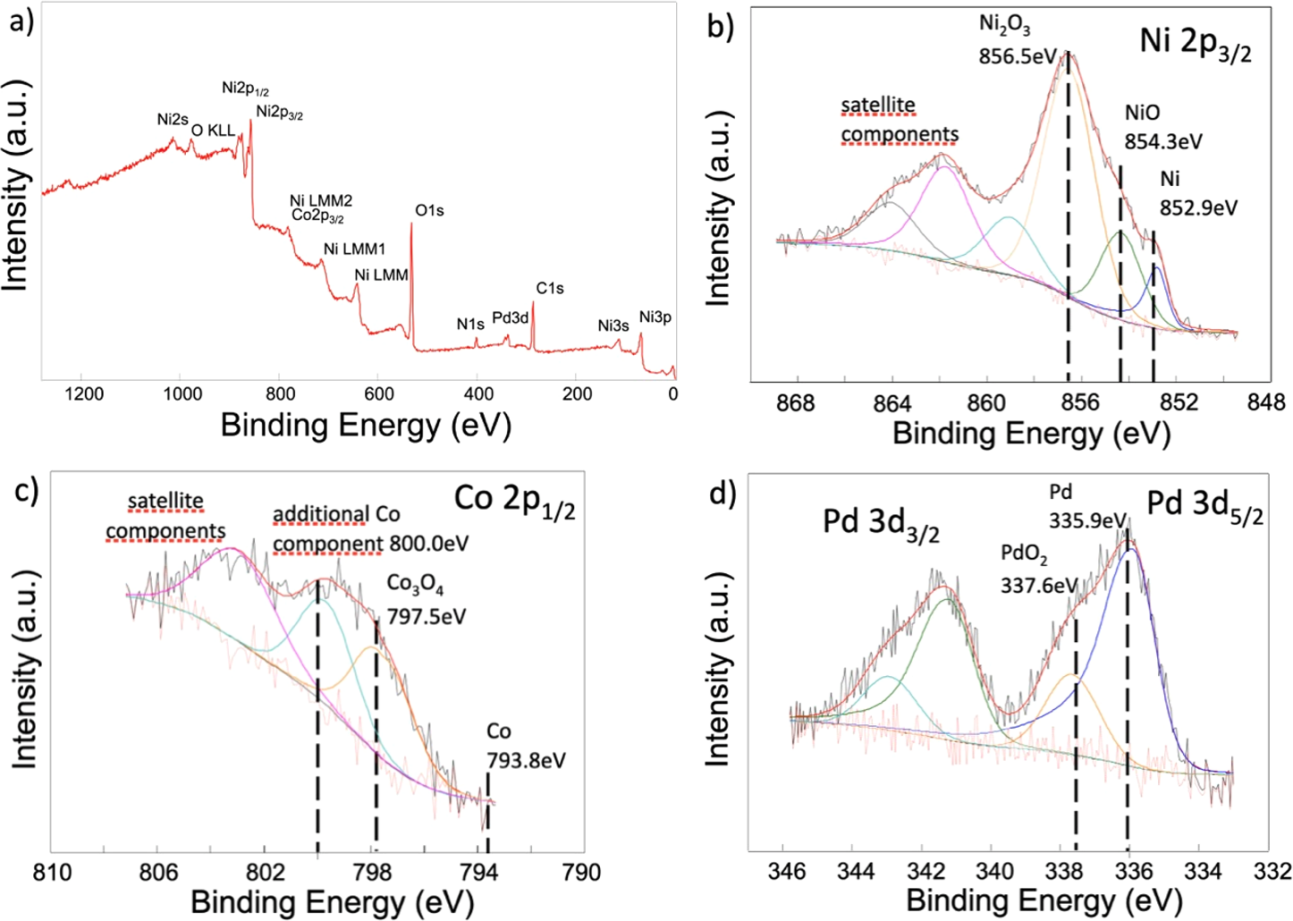
XPS spectra of Pd@Ni_8_–Co_2_: (a) survey spectra and (b) Ni 2p_3/2_, (c) Co 2p_1/2_, and (d) Pd 3d_5/2_ and 3d_3/2_ core levels.

Electrical conductivity studies showed a relationship between the concentration of Co in the structures of the synthesized materials and their conductivity (σ). The conductivity of the Co-rich materials was higher than that of the Ni-rich nanowires, even after the Pd nanoparticle decoration process (Figure 4a,c). The Ni_1_–Co_9_ nanowires had a σ of 5.8 × 10^–4^ S/cm, while that of Pd@Ni_8_–Co_2_ was 1.8 × 10^–4^ S/cm. The observed changes can be related to the differences found on the nanowires’ surface. As confirmed by the XPS analysis, the surface of the samples was oxidized and rich in NiO, Ni_2_O_3_, and Co_3_O_4_ oxides. This oxidized layer also covered samples with a higher Co content. Therefore, the higher conductivity can be related to the much easier electron transport by the Co_3_O_4_ phase and the presence of the mixed valence state of Co ions in Co_3_O_4_, similar to the case for Fe_3_O_4_.^62−64^ Moreover, the electrical conductivity was almost constant for all samples over a wide frequency range (from 0.1 to 1 × 10^5^ Hz), confirming their typical metallic conductivity behavior. This behavior was additionally confirmed by analyzing the phase angle (Φ) value over the wide frequency range used for the conductivity (Figure 4b). This parameter can be easily used to determine the nature of the analyzed sample. If the Φ value equals −90°, the material can be described as a pure capacitor. A positive value of 90° is characteristic of pure inductors, while a phase angle of 0° characterizes pure resistors. As one can see in Figure 4b, all of the samples can be treated as pure resistors at low frequencies, and the behavior changes only at higher frequencies (>10^4^ Hz), when Φ moves slightly into a negative value with an increased frequency (especially for samples with a higher nickel concentration). The deposition of Pd nanoparticles on the surface of Ni_7_–Co_3_ and Ni_8_–Co_2_ nanowires resulted in an increase in their conductivity, but in the second case, the recorded change was significantly greater, from 2.7 × 10^–5^ to 1.8 × 10^–4^ S/cm (Figure 4c).

**Figure 4:**
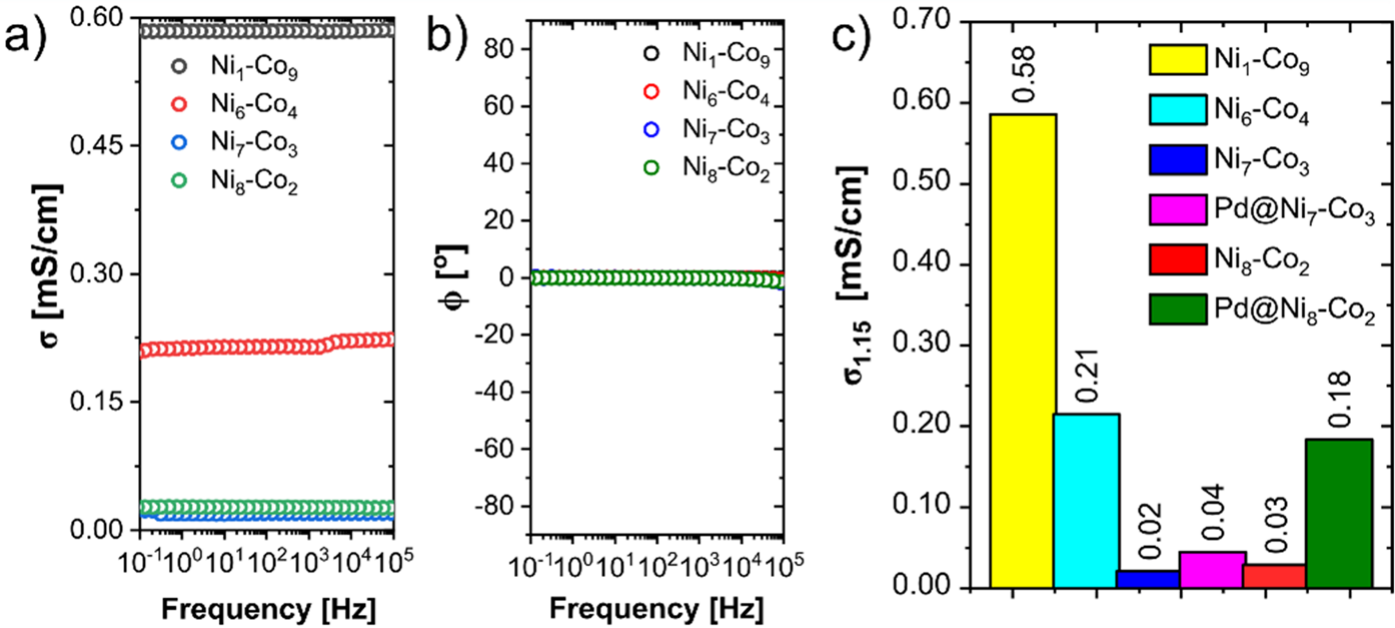
(a) Electrical conductivity of Ni_*x*_–Co_*y*_ nanowires as a function of frequency. (b) Phase angle as a function of frequency measured for Ni_*x*_–Co_*y*_ nanowires. (c) Comparison of the electrical conductivity of Ni_*x*_–Co_*y*_ and Pd@Ni_*x*_–Co_*y*_ nanowires at 1.15 Hz.

The electrochemical activity of Ni_*x*_–Co_*y*_ and Pd@Ni_*x*_–Co_*y*_ nanowires toward the ORR was assessed by obtaining linear sweep voltammetry (LSV) polarization curves recorded at a rotating speed of 1600 rpm (Figure 5a,b). The Ni_*x*_–Co_*y*_ nanowires free of Pd showed poor catalytic activity with an *E*_onset_ of <0.72 V. A higher or equimolar content of Ni with respect to Co in the composition facilitated the ORR. The electrocatalytic activity was significantly enhanced by the deposition of Pd on the surface of the nanowires, indicated by a shift of the onset potential toward positive values [observed for the whole Ni_*x*_–Co_*y*_ series (Figure 5b)]. The onset potential changed in the following order: Pd@Ni_8_–Co_2_ > Pd@Ni_7_–Co_3_ > Pd@Ni_9_–Co_1_ > Pd@Ni_6_–Co_4_ > Pd@Ni_3_–Co_7_ > Pd@Ni_5_–Co_5_ > Pd@Ni_1_–Co_9_. This indicated a higher activity toward the ORR for the nanowires comprising higher loadings of Ni species in the composition. The onset potential for the best-performing Pd@Ni_8_–Co_2_ was approximately 0.89 V. For the materials with higher loadings of Ni (Pd@Ni_8_–Co_2_ > Pd@Ni_7_–Co_3_ > Pd@Ni_9_–Co_1_), the diffusion-limited current was also significantly improved (approximately −5.34 mA cm^–2^). The increase in activity can be ascribed to the superior morphology of the materials, wherein the Ni species facilitate the formation of spikes (Figure 2b,e), suitable supports for deposited Pd that provide improved access to the active sites. Moreover, the created interfaces (SMSI) between the Ni–Co support and the Pd catalyst can boost charge transfer and, therefore, improve the system’s catalytic performance.^13^

**Figure 5:**
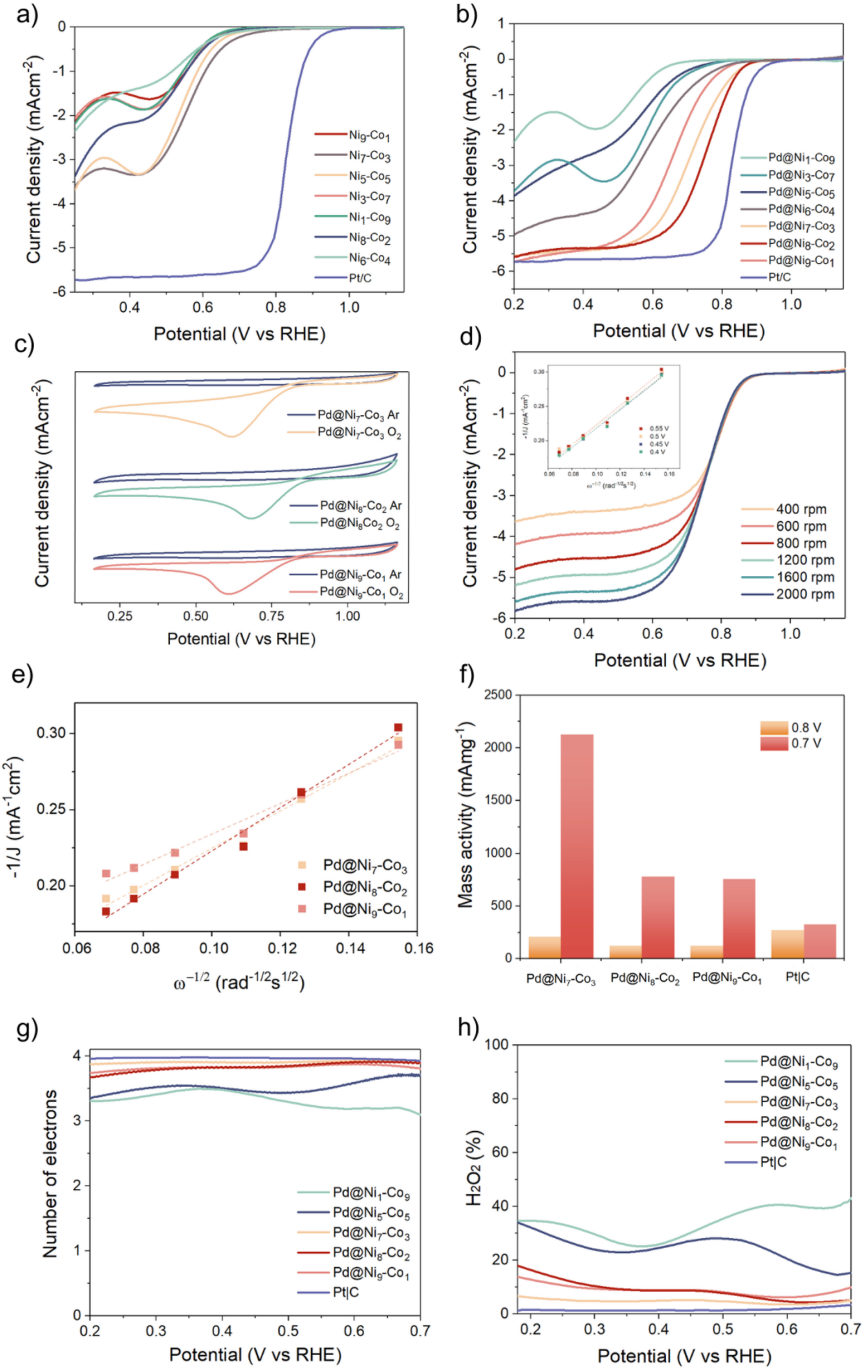
(a) LSVs recorded for the Ni_*x*_–Co_*y*_ nanowires deposited on a GC electrode in O_2_-saturated 0.1 M KOH at a scan rate of 50 mV/s. (b) LSVs recorded for Pd@Ni_*x*_–Co_*y*_ deposited on a GC electrode in O_2_-saturated 0.1 M KOH at a scan rate of 50 mV/s. (c) CV curves recorded in Ar- and O_2_-saturated 0.1 M KOH at a scan rate of 10 mV s^–1^. (d) LSVs recorded for Pd@Ni_8_–Co_2_ in O_2_-saturated 0.1 M KOH at various rotation speeds (the inset image shows the K–L plots at various potentials). (e) Corresponding K–L plots for Pd@Ni_8_–Co_2_, Pd@Ni_7_–Co_3_, and Pd@Ni_9_–Co_1_ at 0.55 V. (f) Corresponding mass activities of the catalysts at 0.70 and 0.80 V. (g) Corresponding electron transfer numbers and (h) peroxide yields (H_2_O_2_%) calculated from the RRDE.

The appreciable electrocatalytic activity of the materials toward the ORR was confirmed by cyclic voltammetry (CV), measured using a three-electrode system in a 0.1 M KOH solution (Figure 5c). In O_2_-saturated solutions, representative Pd@Ni_8_–Co_2_, Pd@Ni_7_–Co_3_, and Pd@Ni_9_–Co_1_ samples showed irreversible cathodic peaks at 0.87, 0.62, and 0.60 V, respectively, associated with the irreversible reduction of oxygen. In contrast, in Ar-saturated solutions, quasi-rectangular voltammograms with no significant redox peak were observed in the potential range of 0.25–1.12 V. To further investigate the electron transfer ability of the materials, rotating ring-disk electrode (RRDE) measurements at various rotation speeds (400–2000 rpm) were performed and the corresponding Koutecky–Levich (K–L) plots were produced (Figure 5d and Figures S11b,c and S12b,c). An increase in electrode rotation speed did not affect the shift in the onset potential in either direction, while an increase in current density was observed, the reason for which may be improved mass transport. The K–L plots obtained from the polarization curves at various potentials for the Pd@Ni_8_–Co_2_ catalyst showed good linearity, indicating the first-order reaction kinetics of the process (Figure 5d, inset, and Figure 5e). The MAs of the catalysts at 0.70 and 0.80 V were determined (referenced to the mass of Pd) and are depicted in Figure 5f. At 0.70 V, Pd@Ni_7_–Co_3_ exhibited the most significant MA (>2000 mA mg^–1^) among the investigated materials. The values were much lower (<750 mA mg^–1^) for Pd@Ni_8_–Co_2_ and Pd@Ni_9_–Co_1_. At 0.80 V, Pd@Ni_7_–Co_3_ still exhibited the largest MA (220 mA mg^–1^) among the investigated samples. However, it was lower than that of commercial Pt/C (260 mA mg^–1^).

For better visualization, the number of electrons transferred was calculated from the ring and disc currents and is depicted in Figure 5g. The electron numbers calculated for the materials with a high Ni content in the composition (Pd@Ni_8_–Co_2_, Pd@Ni_7_–Co_3_, and Pd@Ni_9_–Co_1_) approached four in the potential range of 0.2–0.7 V, indicating the occurrence of the dominant four-electron process (O_2_ to H_2_O), as for the commercial Pt/C catalyst. For the materials with lower Ni contents (Pd@Ni_1_–Co_9_ and Pd@Ni_5_–Co_5_), the electron transfer number was lower (∼3.5), indicating the occurrence of both the four- and two-electron processes (O_2_ to H_2_O_2_). The calculated peroxide yield (H_2_O_2_%) was roughly constant in most cases over the potential range of 0.2–0.7 V, with the lowest (<7%), for Pd@Ni_7_–Co_3_, being close to that of the commercial catalyst (Figure 5h).

Finally, even though the ORR occurred at lower potentials for the investigated materials than for the commercial 20 wt % Pt/C catalyst [onset potential of 0.94 V (Figure 5b)], the investigated hybrid materials showed good methanol tolerance, unlike the commercially available Pt/C catalyst (Figure 6).

**Figure 6:**
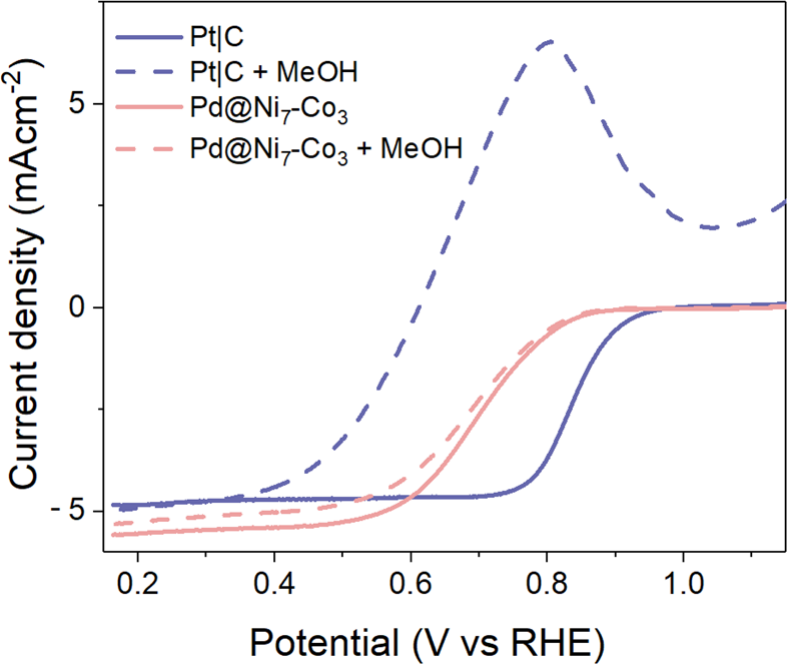
LSV recorded for Pd@Ni_7_–Co_3_ and Pt/C deposited on a GC electrode in 0.1 M KOH with 3 M CH_3_OH and in a 0.1 M KOH solution.

The support material for PGM nanoparticles used in the ORR should be characterized by an appropriately large active surface area, electrical conductivity, crystallinity, and stability. In this study, we validated bimetallic Ni–Co nanowires decorated with Pd nanoparticles as a new platform for ORR. By changing the concentration of the employed Ni–Co precursors in the synthesized supports, we tuned the electrical and electrochemical properties of the produced systems with Pd. Additionally, by combining the Ni–Co transition metal support with Pd nanoparticles, we modulated the electronic structure of the latter through the SMSI mechanism, improving the catalytic activity of the Pd@Ni–Co materials. For the Pd@Ni_8_–Co_2_ system, a significant increase in electrical conductivity from 2.7 × 10^–5^ to 1.8 × 10^–4^ S/cm was recorded before and after the decoration of spherical Pd nanoparticles (∼5 nm) and the best ORR reduction potential value of 0.89 V among the materials produced was recorded, which was slightly lower than that of commercial Pt/C (0.94 V).

On the contrary, the Pd@Ni_7_–Co_3_ system exhibited the highest value of MA, >2000 mA mg^–1^ at 0.7 V and 220 mA mg^–1^ at 0.8 V, whereas the MA of Pt/C was >300 mA mg^–1^ at 0.7 V and 260 mA mg^–1^ at 0.8 V. The material presented good stability during testing with methanol, while the same cannot be said for Pt/C. The properties of materials with a predominance of Ni in the composition are determined by the Ni(Co) phase, which favored the formation of spiky nanowires with a highly developed surface and better catalytic activity. In contrast, systems with a predominance of Co promoted the formation of a hexagonal phase, Co_0.75_Ni_0.25_, which crystallized in the form of spherical beads with a significant degree of surface oxidation and better conductivity. In summary, our research showed not only that the designed materials have application potential for the ORR but also their underlying surface–property interactions. These relationships are essential for the design of other catalytic systems based on transition metals for use in electrochemical reactions.

## Methods

*Synthesis of the Ni–Co support*. The developed procedure for producing metallic supports was based on that described in ref (65). In brief, a 0.1 M solution of NaOH (70 mL) in ethylene glycol was mixed with an aqueous solution of 64% hydrazine (5 mL). The precursor solution (22 mL) was slowly dispensed into the heated (90 °C) mixture. Variable volume ratios of the metal chlorides NiCl_2_ and CoCl_2_ (9:1, 8:2, 7:3, 6:4, 5:5, 3:7, and 1:9) were dissolved in ethylene glycol and mixed with an aqueous solution of 0.01 M H_2_PtCl_6_ (2 mL). The 1D growth of the supports was carried out for 10 min in the presence of a magnetic field (neodymium magnet). The obtained materials were washed several times with distilled water and acetone.

*Synthesis of Pd@Ni–Co Materials*. To an aqueous solution of K_2_PdCl_4_ (100 mL) were added the fabricated metallic Ni–Co supports (120 mg) and PVP (100 mg). The resulting mixture was then heated (60 °C) and sonicated under a protective atmosphere for 1 h. After the process had been completed, the produced Ni–Co@Pd materials were washed with distilled water and acetone.

*Characterization*. Phase analysis of the obtained materials was performed using XRD (Rigaku MiniFlex 600) with a Cu Kα lamp (λ = 0.15406 nm). The morphology and structure of the samples were characterized by using a Titan 80-300 S/TEM microscope. TEM, HRTEM, BF-STEM, and HAADF-STEM images were recorded at 300 kV. Local chemical composition analysis of the samples in the microscope was carried out by using an EDX detector. The electron structure of the materials studied was determined with an XPS spectrometer (PHI5700, Physical Electronics) using Al Kα radiation (*h*ν = 1486.6 eV). XPS measurements were performed directly on the fabricated samples and after they were etched with an Ar^+^-ion beam (1 keV, 1 min). Mass analysis of the chemical composition of the samples was performed on an ICP-OES instrument (Optima 2100 DV, PerkinElmer).

The electrical properties of Ni_*x*_–Co_*y*_ metallic supports before and after Pd deposition were characterized by using a dielectric spectrometer (Concept 81, Novocontrol Technologies) equipped with an Alpha analyzer. For this purpose, the samples were sonicated with ultrapure isopropyl alcohol to form a paste and placed dropwise on a 20 mm interdigitated electrode surface (150 μm spacing distance), also from Novocontrol Technologies. Measurements were performed at room temperature over a frequency range of 10^–2^ to 10^5^ Hz using an ac voltage amplitude with impedance measurements of 10 Vrms without additional dc bias.

The electrochemical activity toward ORR was evaluated using an Autolab PGSTAT 204 instrument. For the rotating ring-disk electrode (RRDE) measurements, a GC-disk electrode with a platinum ring (Autolab, 5 mm diameter) was employed in a standard three-electrode system. For CV measurements, a GC-disk electrode (eDAQ, 1 mm diameter) was used in a similar three-electrode setup. A carbon rod was used as the counter electrode, while a Ag/AgCl electrode served as the reference electrode in a 0.1 M KOH electrolyte, being bubbled with O_2_ for 20 min prior to each experiment. The measured potential was converted into the standard reversible hydrogen electrode (RHE) potential by using the following equation:
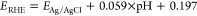
1

To ensure a clean and uniform substrate surface, the GC-disk electrode was polished with alumina and rinsed with deionized water before each experiment. The catalyst ink was prepared by mixing 5.0 mg of the material with 25 μL of aqueous Nafion, 250 μL of ethanol, and 100 μL of water in a glass vial. The mixture was sonicated for 10 min to obtain a well-dispersed, homogeneous solution. A precise and controlled deposition of the catalyst was achieved by applying 5 μL of the ink to the surface of the GC-disk electrode, and the solvent was allowed to evaporate slowly at room temperature under air to obtain a uniform and robust film with a catalyst loading of ∼0.34 mg cm^–2^. As the reference catalyst, commercial 20 wt % Pt/C was used. During the RRDE experiments, the ring potential was held constant at 0.5 V versus the reference electrode. The results of the RRDE experiments were used to determine electron transfer numbers (*n*) and peroxide yields [*Y* (percent)] using eqs 1 and 2, respectively:
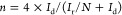
2
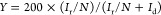
3where *I*_r_ and *I*_d_ are the disk and ring currents, respectively, and *N* is the ring collection efficiency (24.9%). The kinetic parameters for the ORR were calculated by the K–L equation: *J*^–1^ = *J*_K_^–1^ + *J*_L_^–1^ where *J* is the measured current density and *J*_K_ and *J*_L_ correspond to the kinetic and diffusion-limited current densities, respectively.

## Supporting Information Available

Chemical composition of the tested materials and additional structural and electrochemical properties (PDF)
