# Supplementary material for: Tuning the Structure of Pd@Ni–Co Nanowires and Their Electrochemical Properties
Source: J Phys Chem Lett. 2024 Apr 4;15(15):4006–14. doi: 10.1021/acs.jpclett.4c00376 (PMC11033936; doi:10.1021/acs.jpclett.4c00376)
Supplement: Supplementary file 1 — jz4c00376_si_001.pdf [file jz4c00376_si_001.pdf]

Supporting Information for

Tuning the Structure of Pd@Ni-Co Nanowires and Their Electrochemical Properties

Dariusz Łukowiec, [1,*] Magdalena Gwóźdź, [2] Alina Brzęczek-Szafran, [2] Tomasz Wasiak, [2] Dawid Janas, [2] Jerzy Kubacki, [3] Stanisław Wacławek, [4] and Adrian Radoń, [5]

[1] Materials Research Laboratory, Faculty of Mechanical Engineering, Silesian University of Technology, Konarskiego 18A, Gliwice 44-100, Poland,

[2] Faculty of Chemistry, Silesian University of Technology, Krzywoustego 4, Gliwice 44-100, Poland

[3] August Chełkowski Institute of Physics, Faculty of Science and Technology, University of Silesia, 75 Pułku Piechoty 1, Chorzów 41-500, Poland

[4] Institute for Nanomaterials, Advanced Technologies and Innovation, Technical University of Liberec, Studentská 1402/2, Liberec 1 461 17, Czech Republic

[5] Łukasiewicz Research Network - Institute of Non-Ferrous Metals, Sowińskiego 5, Gliwice 44-100, Poland

* Corresponding author. E-mail: dariusz.lukowiec@polsl.pl

Table S1. Chemical composition of Pd@Ni_x-Co_y materials based on inductively coupled plasma - optical emission spectroscopy (ICP-OES).

Sample	Ratio Ni:Co	C K_2PdCl_4 , mM	Ni content, %	Co content, %	Pd content, %
Pd@Ni_9-Co_1	9:1	0.5 mM	88.6	10.7	0.70
Pd@Ni_8-Co_2	8:2	0.5 mM	77.9	19.4	2.70
Pd@Ni_7-Co_3	7:3	0.5 mM	68.81	30.37	0.82
Pd@Ni_6-Co_4	6:4	0.5 mM	60.8	37.66	1.54
Pd@Ni_5-Co_5	5:5	0.5 mM	51.03	48.19	0.78
Pd@Ni_3-Co_7	3:7	0.5 mM	30.24	68.38	1.38
Pd@Ni_1-Co_9	1:9	0.5 mM	10.8	88.38	0.82

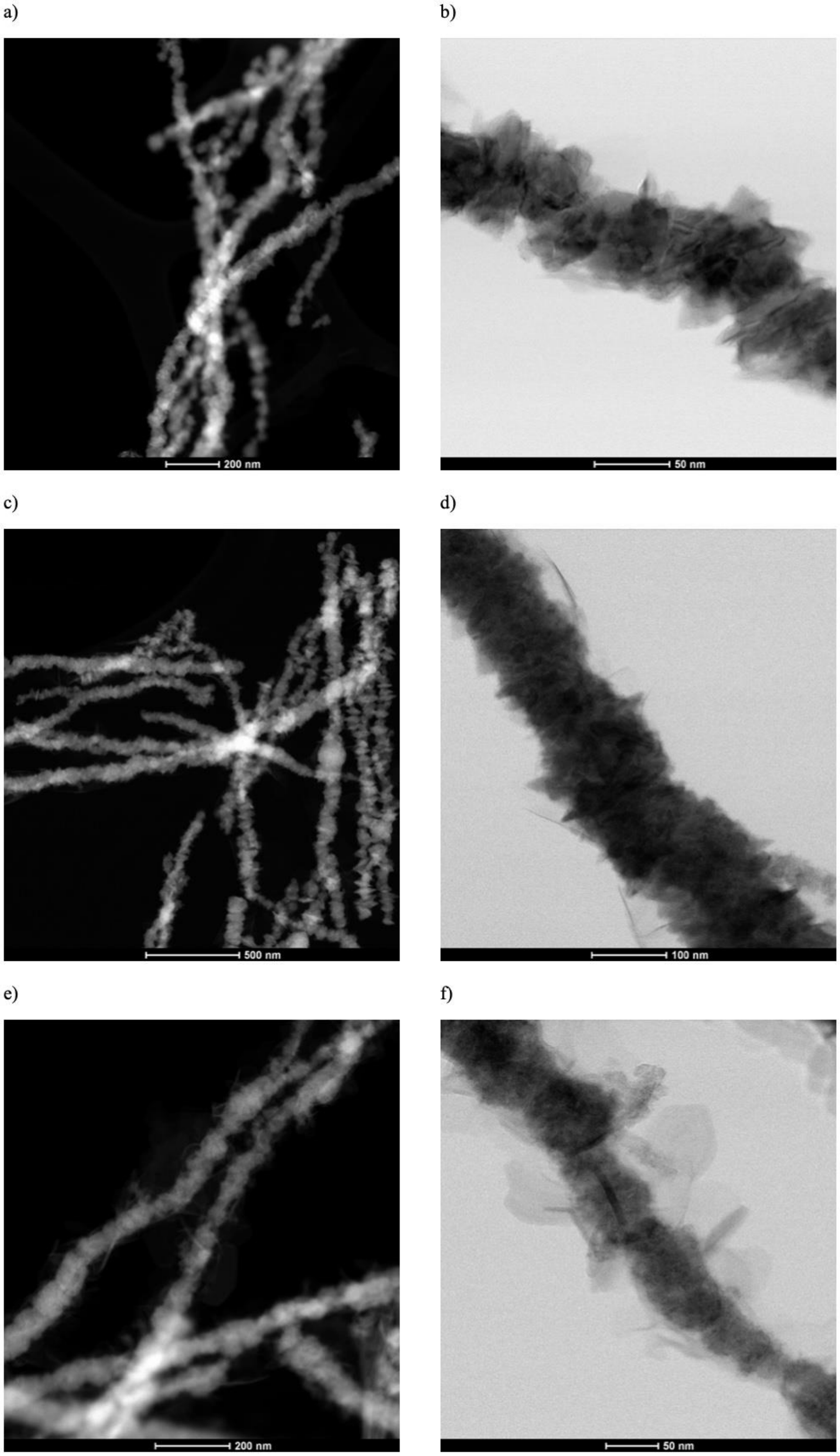

Figure S1. (a) HAADF-STEM and (b) BF-DF STEM images of Pd@Ni_9-Co_1. (c) HAADF-STEM and (d) BF-DF STEM images of Pd@Ni_7-Co_3. (e) HAADF-STEM and (f) BF-DF STEM images of Pd@Ni_1-Co_9.

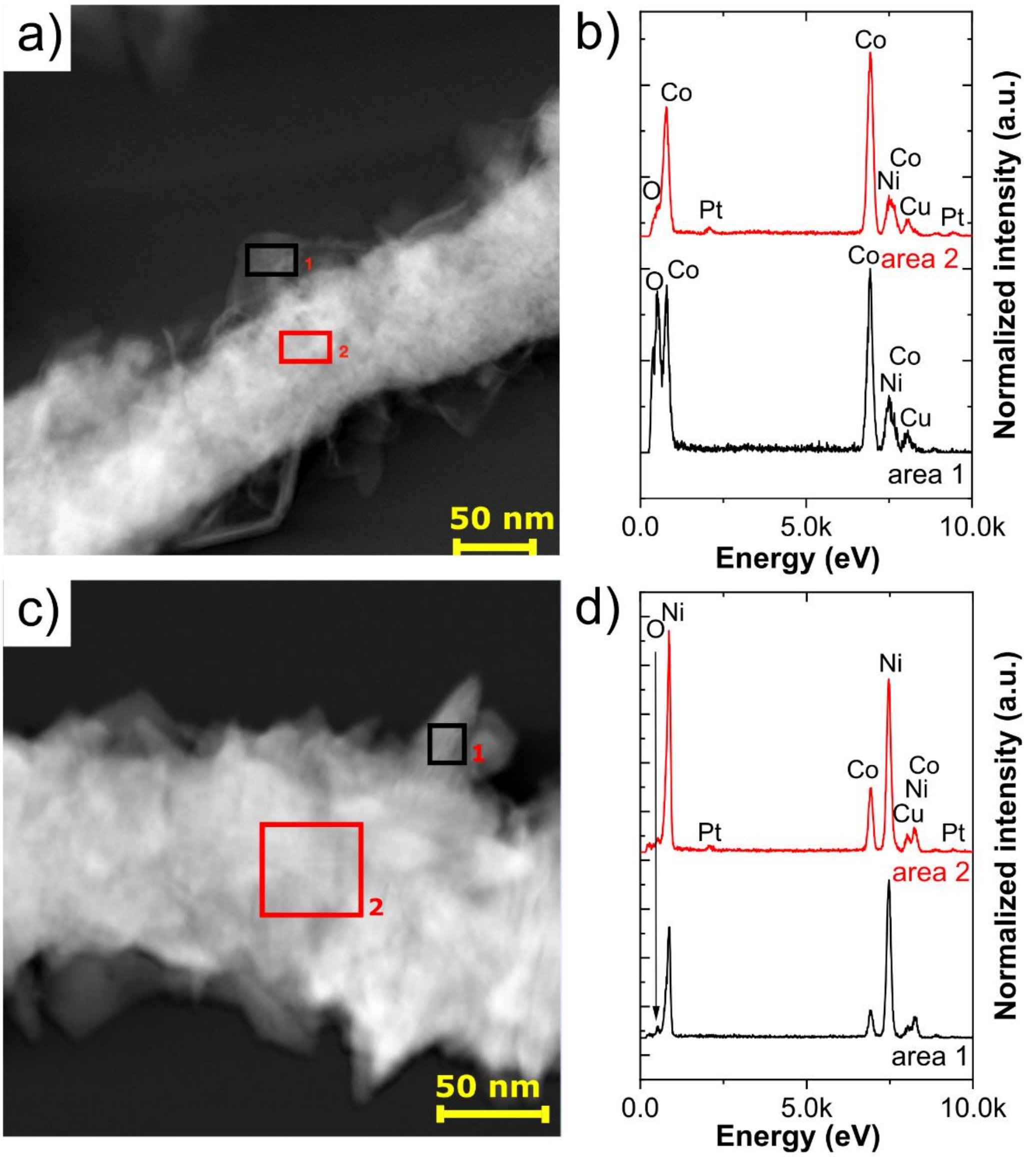

Figure S2. a) HAADF-STEM image of Pd@Ni_3-Co_7 with marked areas of chemical analysis. b) EDX spectra collecting from marked regions 1 and 2 on (a). c) HAADF-STEM image of Pd@Ni_8-Co_2 with marked areas of chemical analysis. d) EDX spectra collecting from marked regions 1 and 2 on (c).

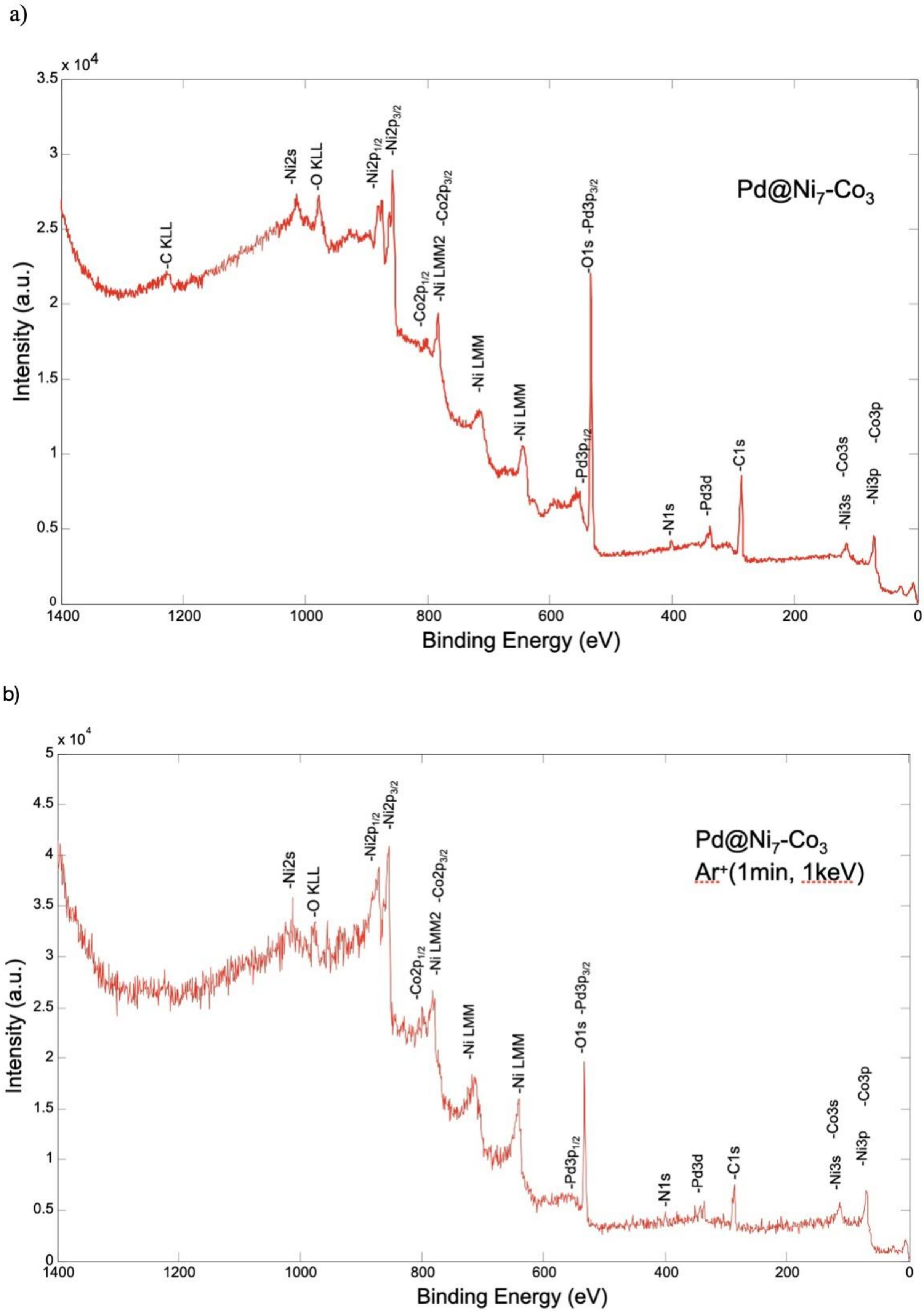

Figure S3. XPS survey spectra of Pd@Ni_7-Co_3: a) before and b) after etching.

a)

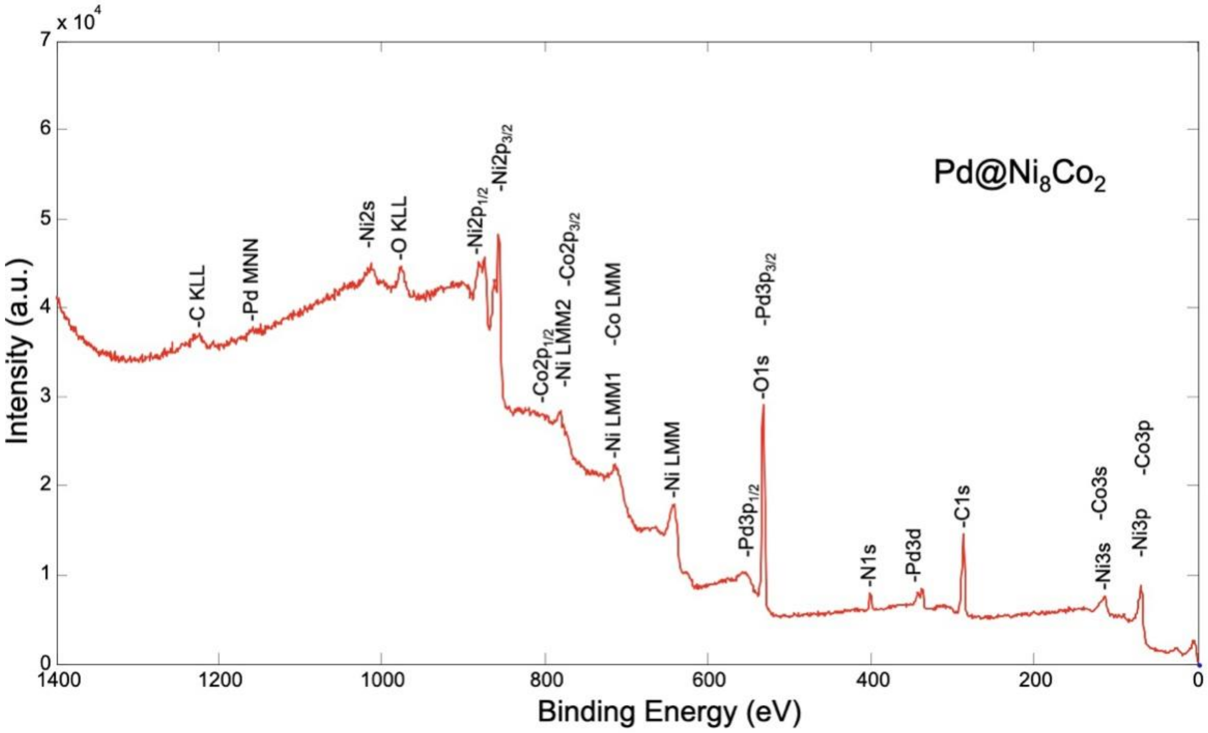

b)

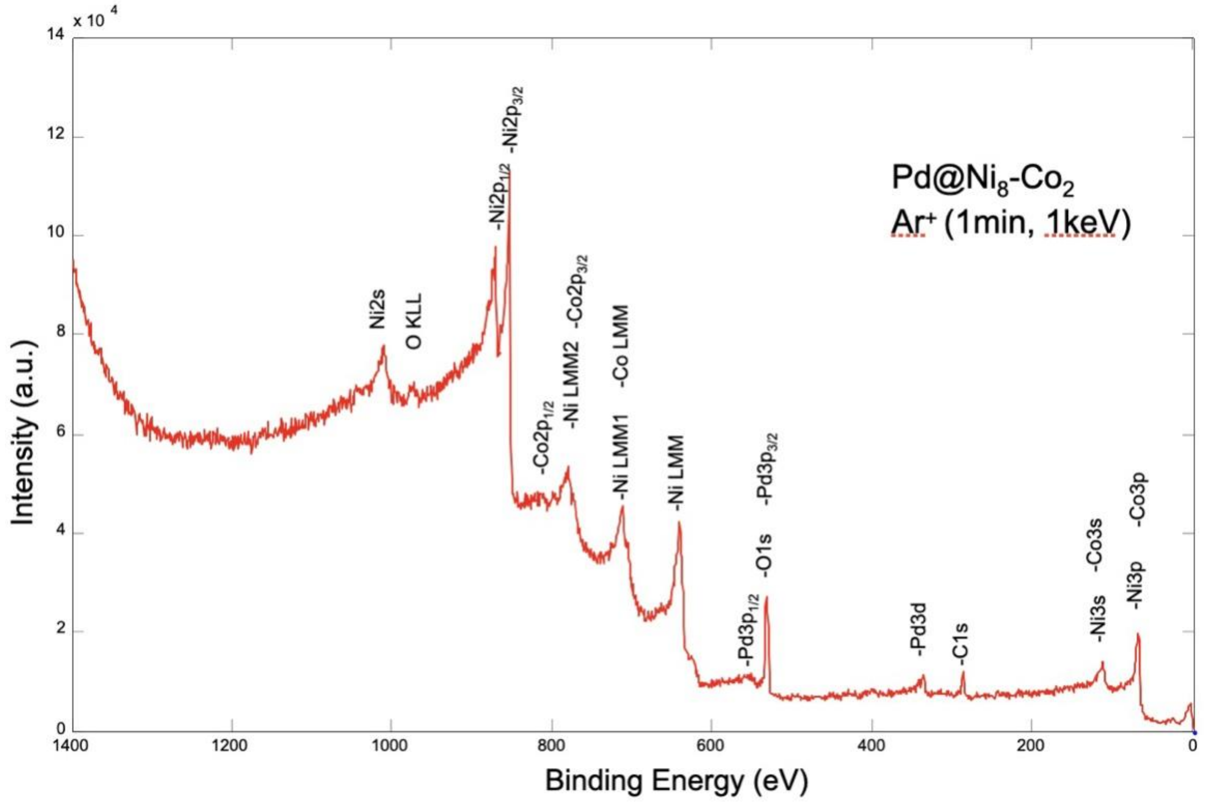

Figure S4. XPS survey spectra of Pd@Ni_8-Co_2: a) before and b) after etching.

a)

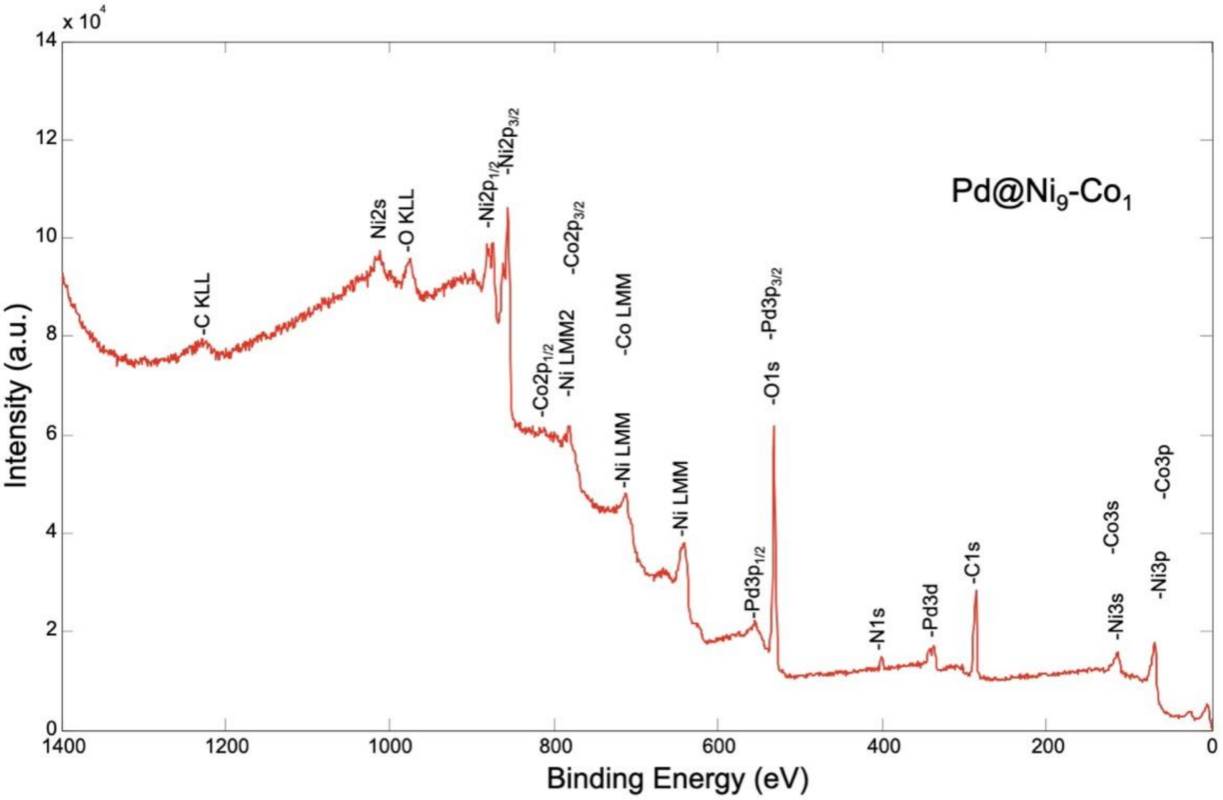

b)

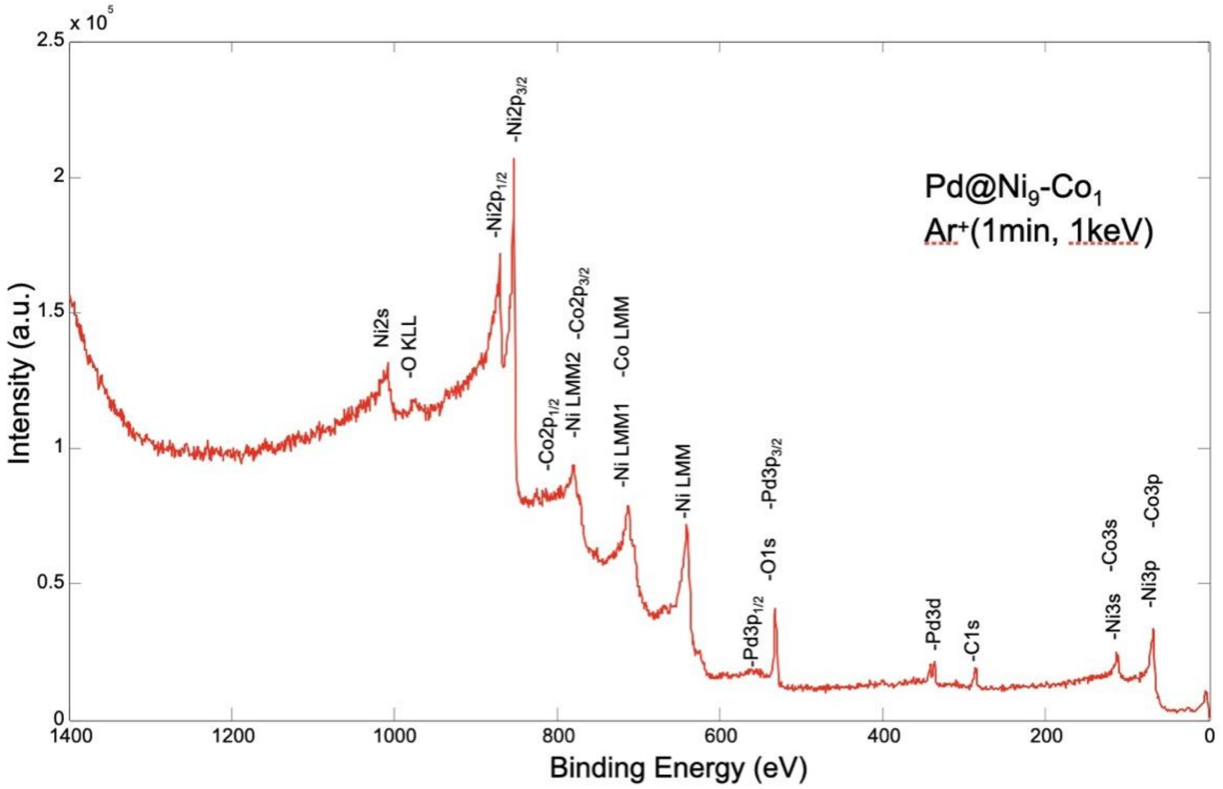

Figure S5. XPS survey spectra of Pd@Ni_9-Co_1: a) before and (b) after etching.

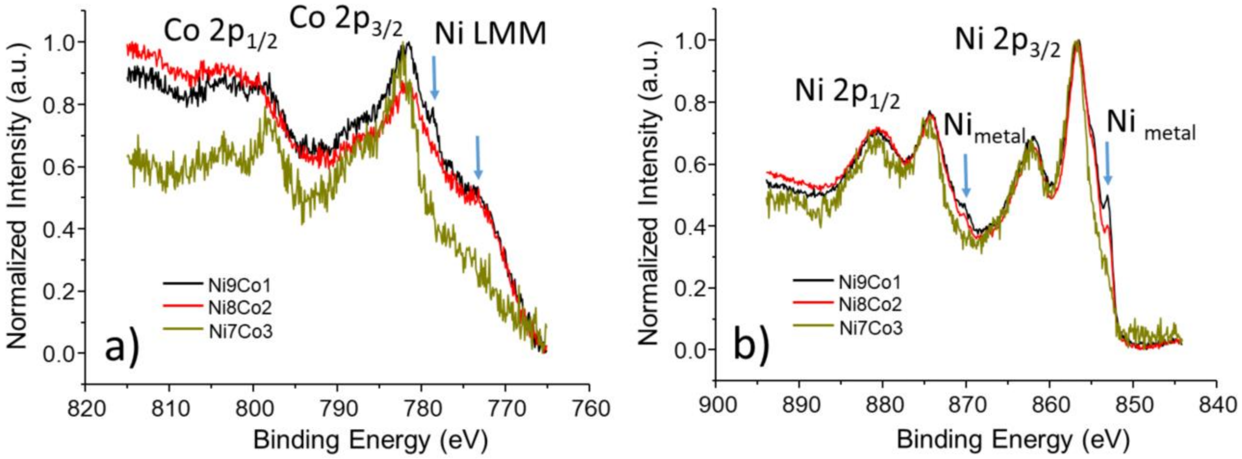

Figure S6. The high-resolution spectra of Co2p (a) and Ni2p (b) doublet with spin orbit coupling $2p_{3/2}$ and $2p_{1/2}$ for the analyzed materials.

The $Co2p_{3/2}$ state is overlapped by the Ni LMM Auger transition. While the shape of the Ni2p doublet only corresponds to the states associated with spin orbit coupling of the $2p_{3/2}$ and $2p_{1/2}$ states, there is a strong overlap between the $Co2p_{3/2}$ photoemission line and the broad Auger transition line of the Ni LMM. The figure below (Fig. S7) shows the overlapping of the Co2p doublet obtained for the Pd@Ni_9Co_1 sample and the broad line associated with the Auger Ni LMM transition (nickel foil, Physical Electronics Company reference spectrum). Therefore, the $Co2p_{1/2}$ state was chosen for the surface chemical state analysis.

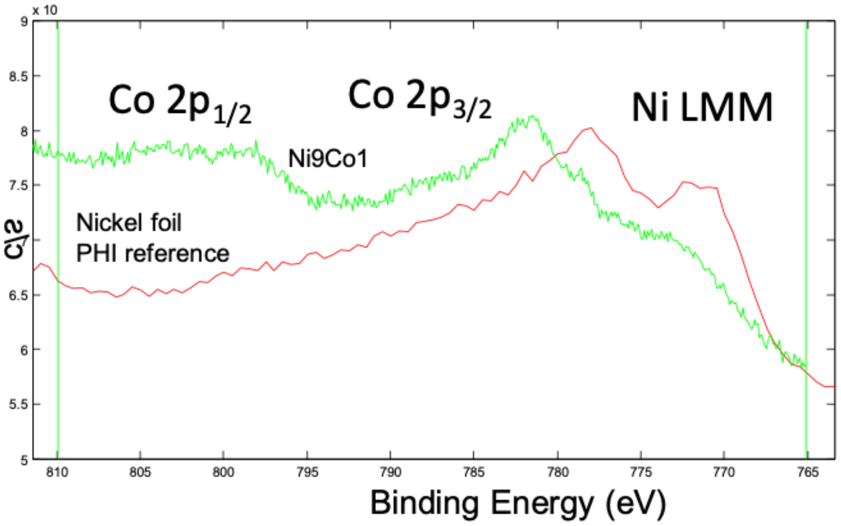

Figure S7. The high-resolution spectra of Co2p (Pd@Ni_9Co_1) and Ni LMM (nickel foil PHI reference).

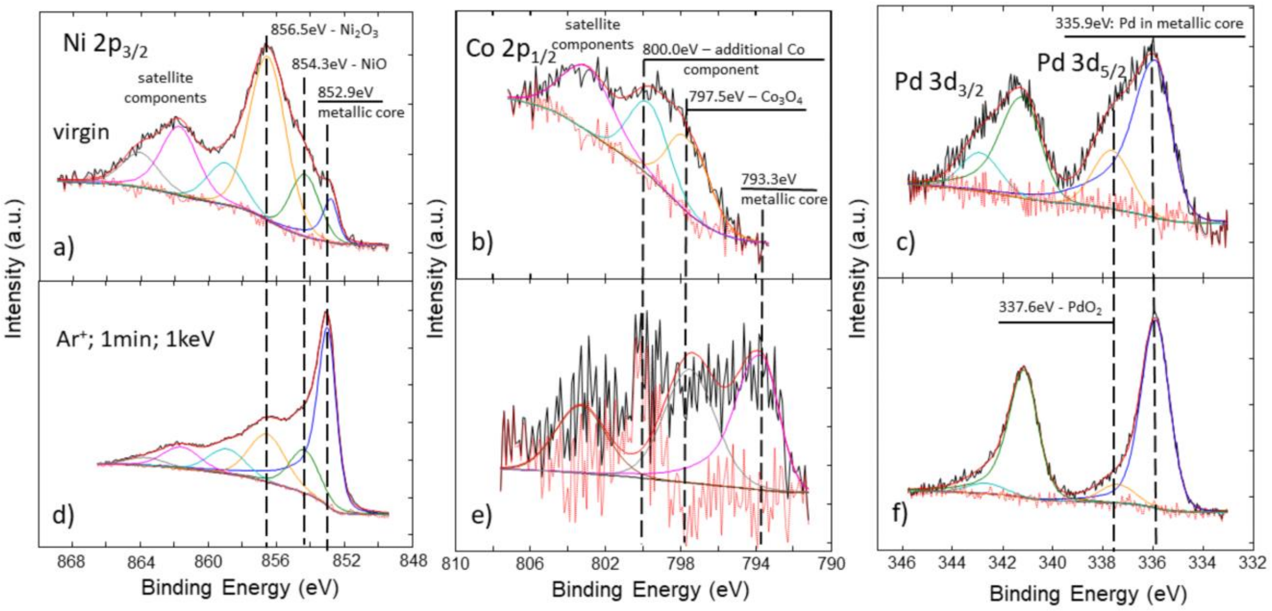

Figure S8. XPS spectra of Pd@Ni_8-Co_2. a) Ni $2p_{3/2}$, b) Co $2p_{1/2}$, c) Pd $3d_{5/2}$ and $3d_{3/2}$ core levels before etching. d) Ni $2p_{3/2}$, e) Co $2p_{1/2}$, f) Pd $3d_{5/2}$ and $3d_{3/2}$ core levels after etching.

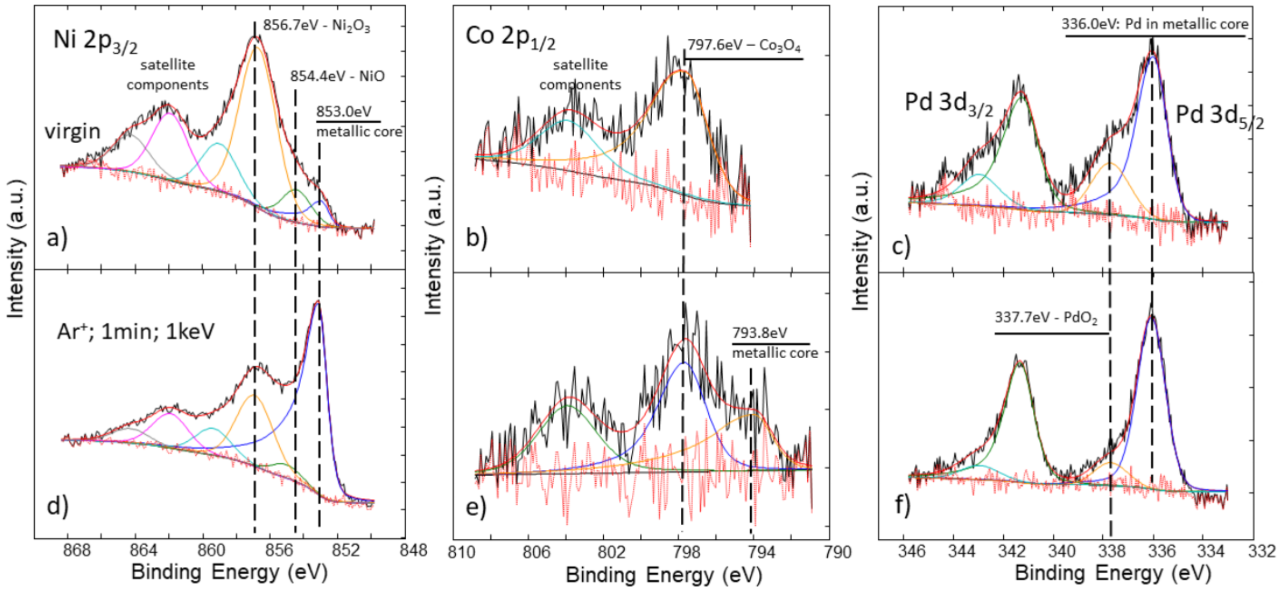

Figure S9. XPS spectra of Pd@Ni_7-Co_3. a) Ni $2p_{3/2}$, b) Co $2p_{1/2}$, c) Pd $3d_{5/2}$ and $3d_{3/2}$ core levels before etching. d) Ni $2p_{3/2}$, e) Co $2p_{1/2}$, f) Pd $3d_{5/2}$ and $3d_{3/2}$ core levels after etching.

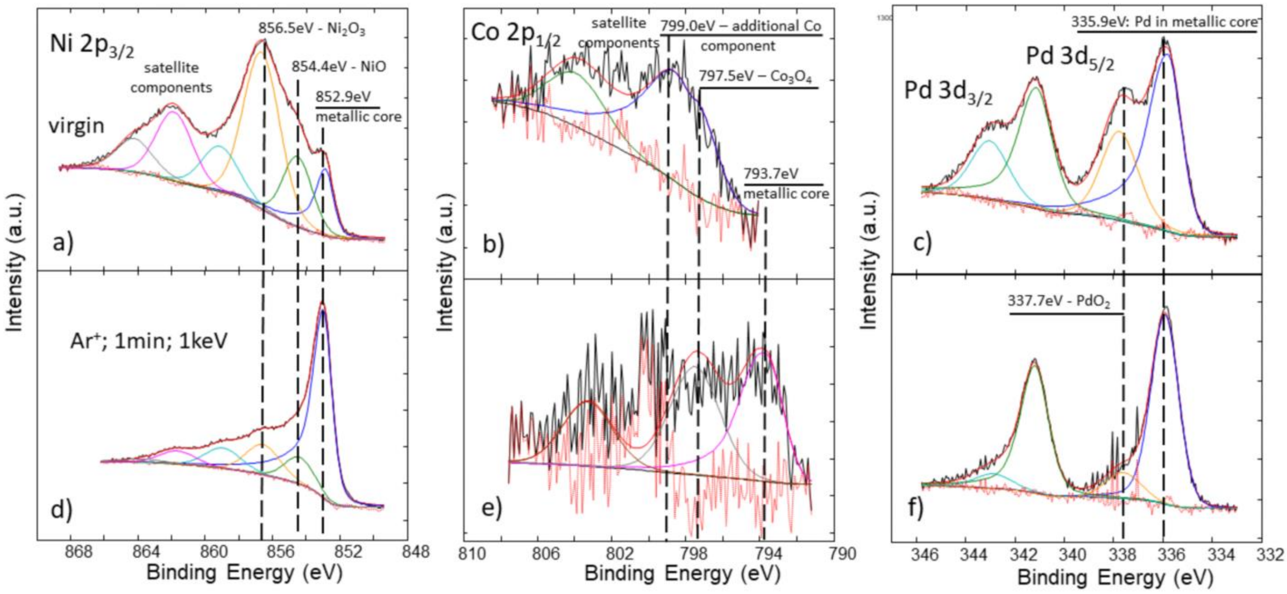

Figure S10. XPS spectra of Pd@Ni_9-Co_1. a) Ni $2p_{3/2}$, b) Co $2p_{1/2}$, c) Pd $3d_{5/2}$ and $3d_{3/2}$ core levels before etching. d) Ni $2p_{3/2}$, e) Co $2p_{1/2}$, f) Pd $3d_{5/2}$ and $3d_{3/2}$ core levels after etching.

Table S2. The atomic and weight concentrations of the cores of the tested materials based on X-ray photoelectron spectroscopy (XPS).

Sample	Atomic/ weight concentration, %	
	Ni2p	Co2p$_{1/2}$
Pd@Ni_9-Co_1	89.3 / 89.2	10.7 / 10.8
Pd@Ni_8-Co_2	82.1 / 82.1	17.9 / 17.9
Pd@Ni_7-Co_3	74.9 / 74.8	25.1 / 25.2

a) b) c)

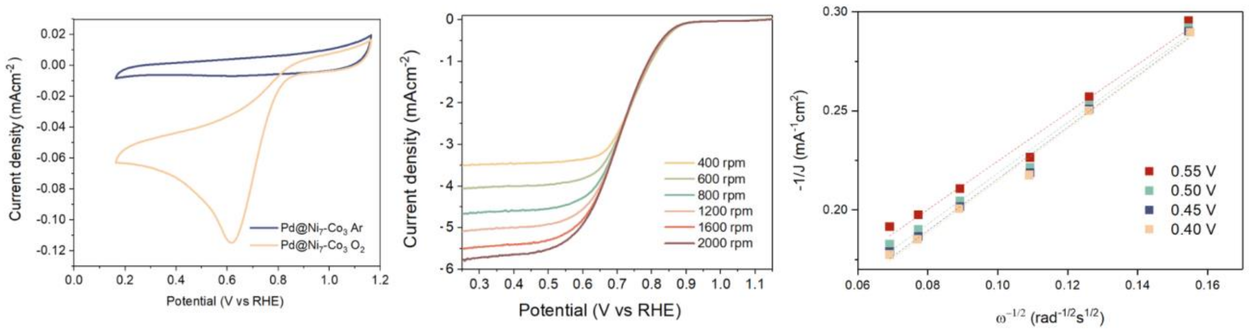

Figure S11. (a) Cyclic voltammetry (CV) curves recorded in Ar- and O_2-saturated 0.1 M KOH at a scan rate of 10 mV s^{-1} for Pd@Ni_7-Co_3@. (b) LSVs recorded for Pd@Ni_7-Co_3 in O_2-saturated 0.1 M KOH at various rotation speeds. (c) The corresponding K-L plots for Pd@Ni_7-Co_3.

a) b) c)

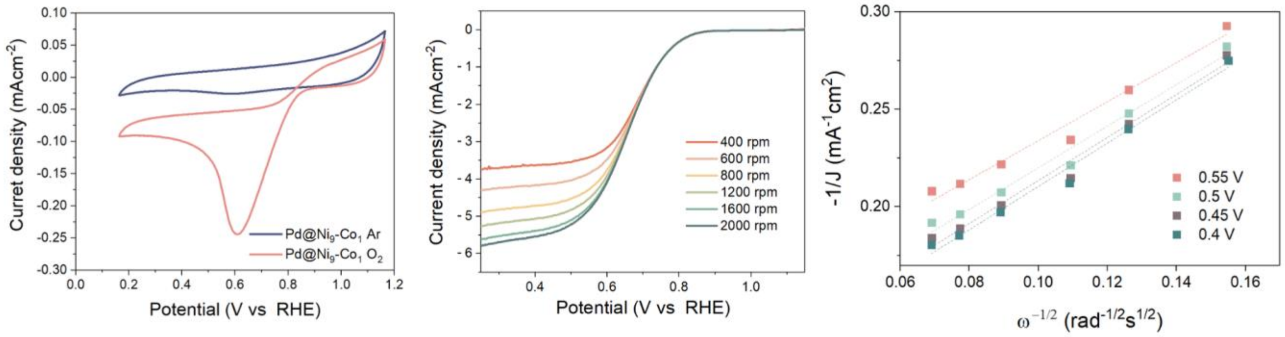

Figure S12. (a) Cyclic voltammetry (CV) curves recorded in Ar- and O_2-saturated 0.1 M KOH at a scan rate of 10 mV s^{-1} for Pd@Ni_9-Co_1. (b) LSVs recorded for Pd@Ni_9-Co_1 in O_2-saturated 0.1 M KOH at various rotation speeds. (c) The corresponding K-L plots for Pd@Ni_9-Co_1.
